# Fluconazole for empiric antifungal therapy in cancer patients with fever and neutropenia

**DOI:** 10.1186/1471-2334-6-173

**Published:** 2006-12-05

**Authors:** Donghui T Yu, Diane L Seger, Josh F Peterson, Ritesh N Kumar, David W Bates

**Affiliations:** 1 Division of General Medicine, Department of Medicine, Brigham and Women's Hospital, Boston, MA, USA; 2 Department of Information Systems, Partners HealthCare System, Boston, MA, USA; 3 Vanderbilt University Medical Center, Nashville, TN, USA; 4 Outcomes Research, Merck & Co., Inc., Whitehouse Station, NJ, USA; 5 Department of Medicine, Harvard Medical School, Boston, MA, USA

## Abstract

**Background:**

Several clinical trials have demonstrated the efficacy of fluconazole as empiric antifungal therapy in cancer patients with fever and neutropenia. Our objective was to assess the frequency and resource utilization associated with treatment failure in cancer patients given empiric fluconazole antifungal therapy in routine inpatient care.

**Methods:**

We performed a retrospective cohort study of cancer patients treated with oral or intravenous fluconazole between 7/97 and 6/01 in a tertiary care hospital. The final study cohort included cancer patients with neutropenia (an absolute neutrophil count below 500 cells/mm^3^) and fever (a temperature above 38°C or 100.4°F), who were receiving at least 96 hours of parenteral antibacterial therapy prior to initiating fluconazole. Patients' responses to empiric therapy were assessed by reviewing patient charts.

**Results:**

Among 103 cancer admissions with fever and neutropenia, treatment failure after initiating empiric fluconazole antifungal therapy occurred in 41% (95% confidence interval (CI) 31% – 50%) of admissions. Patients with a diagnosis of hematological malignancy had increased risk of treatment failure (OR = 4.6, 95% CI 1.5 – 14.8). When treatment failure occurred the mean adjusted increases in length of stay and total costs were 7.4 days (95% CI 3.3 – 11.5) and $18,925 (95% CI 3,289 – 34,563), respectively.

**Conclusion:**

Treatment failure occurred in more than one-third of neutropenic cancer patients on fluconazole as empiric antifungal treatment for fever in routine clinical treatment. The increase in costs when treatment failure occurs is substantial.

## Background

Immunocompromised patients are at risk of contracting serious fungal infections which cause significant morbidity and mortality [1-3]. The diagnosis of fungal infection in immunocompromised cancer patients is difficult for the clinician while the risk of fungal infection is high in patients with prolonged fever and neutropenia who do not receive antifungal therapy [4,5]. Empiric antifungal therapy for patients being treated with broad-spectrum antibiotics who have prolonged fever and neutropenia has been demonstrated to be efficacious, and it has become the standard of practice [5-7]. Newer antifungal agents have used for empiric antifungal therapy included liposomal amphotericin B [8] and caspofungin [9].

Fluconazole is active against the major fungal pathogens (except for mould) in neutropenic cancer patients although conventional amphotericin B is commonly used as a first-line agent for documented infections. Clinical trials have shown that fluconazole is an equally effective but less toxic alternative to amphotericin B for empiric antifungal therapy in cancer patients with prolonged fever and neutropenia [10-13]. These trials also evaluated clinical response to empiric antifungal therapy and found no significant difference in treatment failure with the rates of 25%–44% in fluconazole group compared to 33%–54% in patients on amphotericin B [10,11,13]. The relationships between treatment failure from empiric antifungal therapy and patient characteristics have been explored in a trial [11], although economic outcomes were not reported.

Relatively little information has been available regarding the frequency and resource use of treatment failure using fluconazole as empiric antifungal agent in cancer patients with fever and neutropenia in routine hospital inpatient care. To address these issues, we performed a retrospective cohort study with the goals of: 1) characterizing the patterns of care for cancer patients treated empirically with fluconazole; 2) identifying the rate of treatment failure; 3) assessing the increase in resource utilization associated with treatment failure; and 4) defining the risk factors for treatment failure.

## Methods

### Study site

The study was conducted in a 700-bed tertiary care teaching hospital, Brigham and Women's Hospital, Boston, MA, and has approximately 35,000 admissions per year. Patients were initially identified using the Brigham Integrated Computer System (BICS), which stored patient demographics (age, gender, race, insurance type, etc.) and medication orders. The study was approved by the Partners Human Research Committee.

### Study population

The unit of analysis was the admission. All adult patients admitted and discharged between 7/1/1997 and 6/30/2001, who received oral or intravenous fluconazole were eligible. Cancer patients with fever and neutropenia, and who underwent empiric fluconazole antifungal therapy were included in the final study cohort if they met all of the following inclusion criteria: (1) an oncologic diagnosis; (2) an absolute neutrophil count (ANC) < 500 cells/mm^3 ^within 4 days prior to starting fluconazole; (3) receiving at least 96 hours of parenteral systemic antibacterial therapy; (4) a fever (a temperature > 38°C or 100.4°F) within 4 days prior to start of fluconazole and (5) no documented invasive fungal infections as the indication for initiation of fluconazole (based on the judgment of the care providers as reported in the patients medical record). The second and fourth criteria were referred to any day within the 4 days prior to starting fluconazole. For patients with multiple courses of therapy with fluconazole during the same admission, only those who had neutropenia and fever prior to the first course of fluconazole therapy were included in the final study cohort.

### Data

For patients eligible for the study, data were obtained from both the BICS and the Transition Systems Incorporated (TSI; Boston, MA). For the final study cohort, additional data were collected through a chart review. Demographic and other data including age, gender, race, insurance status (classified as fee-for-service, health maintenance organization, Medicare, Medicaid and none), course of therapy on fluconazole, type of hospital service, primary diagnosis, discharge diagnosis-related group (DRG) and laboratory results including neutrophil count, bacterial and fungal cultures were collected from BICS. Hospital costs were downloaded from the TSI system, which uses a process costing technique to estimate the expenses incurred by cost centers in providing products and services.

In addition, charts were reviewed on the final study cohort to collect additional information for a variety of variables. These included the presence of major underlying diseases, Charlson comorbidity score [14], the presence of organ transplantation, date with fever, information about the use of immunosuppressive therapy before empiric fluconazole therapy, dosage and duration of fluconazole, and presence of antifungal therapy before initiation and after end of the empiric fluconazole therapy. Indications for stopping the empiric fluconazole antifungal therapy were classified as: positive fungal culture one day after starting fluconazole up to the end of course without changing to negative culture, persistent fever at the end of course with negative bacterial and fungal cultures throughout the course of fluconazole, ADE and/or toxicity due to fluconazole, patient death, care withdrawn and planned course of fluconazole completed defined by fluconazole orders having been completed and patients no longer having fever and neutropenia. Information was also collected on whether the patient was discharged on empiric fluconazole therapy or readmitted after being discharged.

### Outcome measures

The primary outcome was treatment failure, which was considered present if any of the following criteria were met: development or persistence of fungal infection evidenced by positive fungal culture during empiric fluconazole therapy and/or up to 7 days after fluconazole was stopped, persistence of fever during empiric fluconazole therapy and/or up to 7 days after fluconaozle therapy without the presence of positive bacterial and fungal cultures, ADE and/or toxicity due to fluconazole, and death from any cause within 7 days after fluconazole was stopped. Positive bacterial and fungal cultures were identified from laboratory reports of tests on specimens from blood, bronchial alveolar lavage fluid, sputum, stool, urine and catheter tips. The date of the laboratory report was used to categorize patients with positive fungal cultures before and after the initiation of empiric therapy. The final study cohort included patients who had positive fungal cultures reported before the initiation of fluconazole, but who did not meet the criteria for diagnosis of invasive fungal infections (no patients had proven or probable invasive fungal infection while one patient had possible invasive fungal infection) as defined by the European Organization for Research and Treatment of Cancer and the National Institute of Allergy and Infectious Diseases Mycoses Study Group [1]. An ADE was defined as "an injury resulting from an intervention related to a drug" [15]. Length of stay (LOS) was measured in days before and after empiric fluconazole antifungal therapy was initiated. Total hospital costs were calculated using a discount rate of 3% annually with the year 2001 as reference. In-hospital mortality was also measured.

### Analyses

For each of the patient characteristics, dosage and duration of fluconazole, and outcomes of measurement, counts with proportions, means with standard deviations, and medians with ranges were determined as appropriate. Univariate comparisons between admissions in which treatment failure developed and those in which it did not were made using the Wilcoxon rank-sum statistic for non-normal continuous variables and the chi-square statistic or Fisher's exact test for categorical variables. To identify independent correlates of treatment failure, multivariate analyses was performed using logistic regression. All variables prior to empiric fluconazole antifungal therapy were included in the baseline model if they had an association with the outcome with a *P *value less than 0.10 in the univariate analysis. Stepwise logistic regression was used to identify correlates of treatment failure, and variables were retained using a threshold of *P *< 0.05. Multivariate comparisons were made using generalized linear modeling, with length of stay and costs as the dependent variable and treatment failure as the main covariate of interest, adjusting for age, gender, insurance status, length of stay before empiric fluconazole therapy, and DRG weight.

A sensitivity analysis was conducted using two approaches. The first approach derived 95 percent confidence intervals using bootstrap estimates (1,000 repetitions) to develop a statistical range for the estimates of total costs and length of stay. With the bootstrap method, one can also determine the stability of the models' effect estimates [16]. The second approach repeated resource utilization models after excluding patients who had positive fungal cultures at baseline because these patients might not be necessarily categorized as on empiric antifungal therapy although none of them had proven invasive fungal infections. Patients who had fungal infections at baseline could have higher resource utilization than others.

The final regression models to predict length of stay and total costs were checked for outliers and influential points [17]. Outliers were identified by examining the jackknife residuals for all patients with studentized residuals greater than 2 in absolute magnitude; six outliers were found. No influential point was detected; the largest value of Cook's distance was 0.18. We therefore present analyses without excluding outliers. All analyses were performed using the SAS statistical package [18].

## Results

Among the total of 1,537 cancer patient admissions in which fluconazole was administered orally and/or intravenously during their hospitalization in the four year study period, 103 (6.7%, 95% CI 5.5% – 8.0%) were treated with empiric fluconazole antifungal therapy for neutropenia and fever after receiving at least 96 hours of parenteral systemic antibacterial therapy. Among these 103 patient admissions, empiric antifungal treatment failed in 42 (40.8%, 95% CI 31.3% – 50.3%) admissions (Table 1). The most frequent causes of treatment failure were positive fungal cultures (20 patients) and persistence of fever with negative bacterial and fungal cultures (19 patients) during empiric fluconazole treatment or within 7 days after the end of empiric therapy. Other causes included fluconazole-associated hepatotoxicity (n = 1) and death earlier than 7 days after the end of empiric fluconazole therapy (n = 5). Three patients had multiple causes of treatment failure. Among the 20 patients with positive fungal cultures after the initiation of empiric therapy, 7 had positive fungal cultures from the same type of specimen prior to beginning empiric therapy.

**Table 1:** Measure of Treatment Failure

**Variable, No. (%)**	**All** **n = 103**
Treatment failure	42 (40.8)
Reason for treatment failure *	
Positive fungal cultures **	20 (19.4)
During empiric fluconazole therapy	16 (15.5)
From day 1 to 7 after fluconazole stopped	6 (5.8)
Persistent fever with negative bacterial and fungal cultures †	19 (18.5)
During empiric fluconazole therapy	17 (16.5)
From day 1 to 7 after fluconazole stopped	4 (3.9)
ADE/toxicity due to fluconazole	1 (1.0)
Death within 7 days after fluconazole stopped	5 (4.9)

ADE, adverse drug event; * Three patients had multiple major reasons including 1 patient had positive fungal culture within 7 days after fluconazole stopped and persistent fever with negative bacterial and fungal cultures during empiric therapy, and 2 patients had persistent fever after initiation of fluconazole and died within 7 days after the end of fluconazole therapy; ** Positive fungal cultures referred to positive tests on specimen from blood, bronchial alverolar lavage, sputum, stool, urine and catheter tips and no report of negative results for repeated test on specimen from the same site, † fever present at least on the last day of fluconazole course and/or on day 7 after fluconazole stopped.

### ADE case report

One treatment failure was due to a serious ADE in a 28-year-old woman with recurrent Hodgkin's disease who had been admitted to the hospital for high dose chemotherapy and bone marrow transplantation. Antifungal treatment was started with intravenous fluconazole 200 mg daily on hospital day 9. On day 12, values of liver function test were elevated (ALT = 147 IU/L, AST = 205 IU/L and BILT = 2.0 mg/dL). Fluconazole was discontinued on day 13. The liver function results returned to a normal value on day 15 and remained normal until the day of discharge. The patient did have chronic active hepatitis B and was on lamivudine but the dose was unchanged during the hospital course.

### Characteristics

The final cancer patient cohort on empiric fluconazole antifungal therapy (Table 2) had a mean age of 51 (+/- 14) years, and was 60% female and 95% white. All patients were receiving care from the oncology or bone marrow transplant services prior to fluconazole treatment. Over three quarters of the patients (76.7%) had hematological malignancies including leukemia and myelodysplastic syndrome (45.6%), lymphoma (22.3%), myeloma (4.9%) and Hodgkin's disease (3.9%), while the remainder of the patients (23.3%) had solid tumors including breast cancer (20.4%), endometrial cancer, neuroendocrine cancer and ovarian cancer. The vast majority of patients (96.1%) were on immunosuppressive therapy with steroids, cyclosporine or chemotherapy and more than half had undergone bone marrow transplantation before fluconazole treatment.

**Table 2:** Patient Characteristics

	**All** **(n = 103)**	**Treatment Failure** **(n = 42)**	**No Treatment Failure** **(n = 61)**
Age, year, mean (SD)	51 (14.1)	51 (14.3)	51 (14.0)
Female, No. (%)	62 (60.2)	24 (57.1)	38 (62.3)
Nonwhite, No. (%)	5 (4.9)	4 (9.5)	1 (1.6)
Uninsured or Medicaid, No. (%)	12 (11.7)	6 (14.3)	6 (9.8)
Primary diagnosis, No. (%)			
Hematological malignancies	79 (76.7)	38 (90.5)	41 (67.2) ‡
Acute leukemia/MDS	40 (38.8)	20 (47.6)	20 (32.8)
Chronic leukemia	7 (6.8)	3 (7.1)	4 (6.6)
Lymphoma	23 (22.3)	11 (26.2)	12 (19.7)
Myeloma	5 (4.9)	3 (7.1)	2 (3.3)
Hodgkin's disease	4 (3.9)	1 (2.4)	3 (4.9)
Solid tumors	24 (23.3)	4 (9.5)	20 (32.8)
Underlying disease, No. (%)	11 (10.7)	5 (11.9)	6 (9.8)
Cardiovascular disease	4 (3.9)	2 (4.8)	2 (3.3)
Others *	7 (6.8)	3 (7.1)	4 (6.6)
DRG weight, mean (SD)	7.4 (3.7)	7.0 (3.6)	7.7 (3.7)
Charlson score, mean (SD)	3.0 (1.7)	2.6 (1.4)	3.3 (1.8) ‡
Charlson score ≤ 4, No. (%)	80 (77.7)	38 (90.5)	42 (68.9) ‡
Treatment before fluconazole started, No. (%)			
Steroid	17 (16.5)	7 (16.7)	10 (16.4)
Chemotherapy	98 (95.2)	38 (90.5)	60 (98.4)
Cyclosporin	6 (5.8)	3 (7.1)	3 (4.9)
History of Transplant, No. (%)			
Bone marrow transplant	56 (54.4)	23 (54.7)	33 (54.1)
Lab results before fluconazole started, No. (%)			
ANC ≤ 100/mm^3^	74 (71.8)	33 (78.6)	41 (67.2)
Positive fungal cultures **	19 (18.5)	9 (21.4)	10 (16.4)
*Candida albicans*	15 (14.6)	8 (19.1)	7 (11.5)
Yeast species	7 (6.8)	3 (7.1)	4 (6.6)
Duration before fluconazole started, day, mean (SD)			
Fever	7.9 (6.8)	7.5 (6.9)	8.3 (6.8)
Neutropenia	6.5 (6.9)	6.5 (6.8)	6.4 (7.0)
Deceased, No. (%)	8 (7.8)	6 (14.3)	2 (3.3) †

MDS, myelodysplastic syndrome; SD, standard deviation; DRG, diagnosis-related group; ANC, absolute neutrophil count;

* Other underlying diseases included chronic pulmonary disease, connective tissue disease, ulcer disease, mild liver disease, mild to moderate diabetes;

** Positive fungal cultures were reported for tests on specimen collected from sputum, stool and urine; three patients had multiple positive cultures from different type of specimen;

† 0.05 ≤ *P *< 0.10, ‡ 0.001 <*P *< 0.05 from Wilcoxon statistic, chi-square statistic or Fisher's exact test for comparision between non-failure and failure groups.

Among 98 patients on chemotherapy before fluconazole was started, 37 patients were on chemotherapy for acute leukemia, including 17 treated with induction chemotherapy and 20 on consolidation chemotherapy. A higher prevalence of positive fungal cultures after initiation of empiric fluconazole therapy was found among patient on induction chemotherapy compared with those on consolidation chemotherapy (24% vs. 5%, *P *= 0.10).

Among 19 patients who had positive fungal cultures before starting fluconazole therapy, isolates included *C. albicans *(15) and yeast species not further identified (7) from stool, urine and sputum specimen; 3 patients had multiple positive fungal cultures from different types of specimens. A total of 8 (7.8%) patients died in hospital with higher prevalence in treatment failure group compared with in non-treatment failure group (14% vs. 3%). There were 5 patients in the treatment failure group who died during empiric therapy or within 7 days after the end of therapy, while 1 patient with and 2 patients without treatment failure died after having been off treatment for more than 7 days. Among those 5 dead in the treatment failure group, 3 were died of sepsis or pneumonia.

### Empiric antifungal therapy

In the 103 admissions (Table 3), all patients received empiric fluconazole antifungal therapy for at least 4 days (average 13.1 days) after admission to the hospital. Among 5 patients on antifungal agent before empiric fluconazole therapy, all were on amphotericin for persistent fever. Regarding fluconazole dosing, 85% (n = 87) were given a maximum daily dosage of 100–200 mg. The mean duration on empiric fluconazole therapy was 6.1 days. The planned course of empiric fluconazole antifungal therapy was completed in 41 (39.8%) admissions; fluconazole was continued after discharge from the hospital in 11% of patients (n = 11). The therapy was discontinued in 50% of admissions (n = 51) (49.5%) admissions, because of treatment failure (36), care withdrawal (1) and in other cases without a specified reason (14). Among the 11 patients discharged on fluconazole, 9 patients were readmitted within 6 months after discharge, including 3 patients with fever and neutropenia but no sufficient information was present to determine whether they experienced fluconazole treatment failure while other 6 patients were readmitted for bone marrow transplant or chemotherapy.

**Table 3:** Empiric Fluconazole Antifungal Therapy

**Variable, No. (%)**	**All** **(n = 103)**	**Treatment Failure** **(n = 42)**	**No Treatment Failure** **(n = 61)**
LOS before fluconazole started, day			
Mean (SD)	13.1 (7.2)	12.7 (6.9)	13.4 (7.4)
Median (range)	12 (4–44)	11 (4–40)	12 (4–44)
On other systemic antifungal agent before fluconazole started			
Amphotericin B	5 (4.9)	0	5 (8.2) †
Total fluconazole dosage, gm			
Mean (SD)	1.2 (0.9)	1.0 (0.6)	1.2 (1.0)
Median (range)	1.0 (0.1–4.4)	0.9 (0.2–2.6)	1.0 (0.1–4.4)
Maximum fluconazole dosage per day, mg			
Mean (SD)	218 (93)	229 (99)	211 (88)
100 – 150 mg	16 (15.5)	4 (9.5)	12 (19.7)
200 mg	71 (68.9)	31 (73.8)	40 (65.6)
400 – 600 mg	16 (15.5)	7 (16.7)	9 (14.8)
Duration on fluconazole, day			
Mean (SD)	6.1 (4.8)	5.3 (3.5)	6.7 (5.4)
Median (range)	5 (1–24)	4 (1–19)	5 (1–24)
Therapy after stopping fluconazole			
Amphotericin B	35 (34.0)	25 (59.5)	10 (16.4) ‡
Another azole angifungal agent	0		
Empiric fluconazole therapy			
Continued after discharged	11 (10.7)	0	11 (18.0) ‡
Stopped before discharged	92 (89.3)	42 (100%)	50 (82.0)
Indication for stopping fluconazole			
Treatment failure	36 (35.0)	36 (85.7)	0 ‡
Positive fungal culture after starting fluconazole *	16 (15.5)	16 (38.1)	0 ‡
Persistent fever after starting fluconazole with negative bacterial and fungal cultures	17 (16.5)	17 (40.5)	0 ‡
ADE/toxicity due to fluconazole	1 (1.0)	1 (2.4)	0
Patient died	2 (1.9)	2 (4.8)	0
Planned course completed	41 (39.8)	1 (2.4) **	40 (65.6) ‡
Care withdrawn/non-specified	15 (14.6)	5 (11.9) **	10 (16.4)

LOS, length of hospital stay; SD, standard deviation; ADE, adverse drug event; * Positive fungal culture included fungal tests on specimen from blood, bronchial alverolar lavage, sputum, stool, urine and catheter tip; ** treatment failure determined as presence of positive fungal culture or persistent fever with negative bacterial and fungal cultures after stopping empiric fluconazole therapy up to 7 days; † 0.05 ≤ *P *< 0.10, ‡ 0.001 ≤ *P *< 0.05 from Wilcoxon statistic, chi-square statistic or Fisher's exact test for comparision between non-failure and failure groups.

### Correlates of treatment failure

Univariate correlates of treatment failure in variables available prior to or during the course of fluconazole therapy were diagnosis of hematological malignancies (*P *= 0.01) and Charlson score ≤ 4 (*P *= 0.01). In the multivariate analysis, the only significant correlate of treatment failure was a diagnosis of hematological malignancy (Odds Ratio = 4.6, 95% CI, 1.5 to 14.8; Table 4).

**Table 4:** Odds Ratios for Predictors of Treatment Failure

Predictors	Odds Ratios (95% CI)	*p*
Hematological malignancies *	4.63 (1.45 – 14.79)	0.01
Charlson score ≤ 4	4.30 (1.34 – 13.76)	0.01

CI, confidence interval;

* The only independent predictor remained in the final logistic regression model using stepwise selection procedure, while the baseline model including hematological malignancies, Charlson score ≤ 4

### Resource utilization of treatment failure

In crude analyses (Table 5), the mean length of stay (mean ± SE) for admissions in which treatment failure occurred was 30.2 ± 1.9 days, versus 23.6 ± 1.5 days for admissions without treatment failure, for a mean difference of 6.6 days (95% CI 1.9 – 11.3). For total costs, the mean difference was $15,888 (95% CI -493 – 32,270). We subsequently performed multiple regression analyses, adjusting for potential confounders including age, gender, insurance status, DRG weight and length of stay before initiating empiric fluconazole therapy. In these analyses, the mean increase in length of stay associated with treatment failure was 7.4 days (95% CI 3.3 – 11.5, *P *= 0.001). For total costs, the mean adjusted increase associated with treatment failure was $18,925 (95% CI 3,289 – 34,563, *P *= 0.02).

**Table 5:** Resource Utilization among Cancer Patients with Empiric Fluconazole Antifungal Therapy by Treatment Failure

	Treatment Failure	No Treatment Failure	Difference	*P*	*R*^2^
N	42	61			
LOS, day					
Unadjusted Mean (SE)	30.3 (1.9)	23.7 (1.5)	6.6	0.007	
Median (range)	27 (8–73)	22 (4–55)	5.0		
Adjusted Mean (SE) *	30.7 (1.6)	23.4 (1.3)	7.3	0.001	0.34
Total Costs per patient, $					
Unadjusted Mean (SE)	71,122 (6,432)	55,234 (5,337)	15,888	0.060	
Median (range)	56,924 (10,647–275,130)	47,642 (3,416–268,447)	9,282		
Adjusted Mean (SE) *	72,921 (6,118)	53,996 (5,068)	18,925	0.020	0.18

LOS, length of stay; SE, standard error;

* Adjusted for age, gender, insurance status (uninsured or Medicaid), DRG weight and length of stay before empiric fluconazole therapy.

After excluding 19 patients who had positive fungal cultures at baseline, we repeated the above analyses comparing the resource utilization between patients with and without empiric antifungal treatment failure (33 vs. 51); the adjusted mean increase in length of stay associated with treatment failure was 8.1 days (95% CI 3.4 – 12.8, *P *= 0.001), and the adjusted mean difference in costs was $21,554 (95% CI 3,034 – 40,074, *P *= 0.03).

In the bootstrap validations, the adjusted mean total costs associated with treatment failure was $18,780 (95% CI 2,515 – 35,044) and the adjusted mean increase in length of stay associated with treatment failure was 7.4 days (95% CI 3.1 – 11.7). We also evaluated the stability of the parameter estimates for both models including all covariates, and the parameter estimates and standard errors were stable for all covariates (data not shown).

## Discussion

In our study population, treatment failure occurred in more than one-third of cancer patients receiving empiric fluconazole antifungal therapy during an episode of febrile neutropenia. This was usually manifested by development and persistence of microbiologically documented fungal infections or persistence of fever. Patients were at higher risk if they had hematological malignancies. Treatment failure was associated with substantially increased length of stay and hospital costs, even after adjusting for potential confounders.

The treatment failure rate found in the current study is in the range of treatment failure or non-success rate (25%–55%) in the fluconazole treatment arms from prospective randomized trials comparing the efficacy of fluconazole and amphotericin B as empiric antifungal agents among cancer patients with neutropenia and fever [10-13,19]. Generally, treatment response was measured similarly in these studies, but there was variability in the length of observation. In each previous trial, evaluation of response was performed by primarily examining the change of fever (with different cut-of-point from 37.6°C to 38.5°C) and clinical or microbiological evidence of fungal infection, development of adverse drug event or death resulting in termination of antifungal medications. As noted above, the follow-up period varied between trials; for example, one trial observed patients response until 7 days after initiation of antifungal medication [10,12], while another followed patients up to 21 days after the completion of antifungal therapy [13]. In this study, we assessed for presence of fungal infection after initiation of empiric fluconazole therapy based on microbiological evidence, and assessed the response to antifungal treatment during and after the end of therapy for 7 days, which was also applied in the most recent large trial [9] to evaluate the efficacy of empiric antifungal therapy in patients with fever and neutropenia.

Surviving for seven days was one of the criteria for successful empiric antifungal treatment [8,9,20]; all deaths after stopping antifungal therapy was classified as treatment failure in previous trials [10,11] and in a recent forum report issued by experts from multiple institutes [21]. This was done because the exact cause of death is often difficult to determine in this setting.

Our findings are partially consistent with previous trials showing that the most frequent causes of treatment failure were positive fungal cultures and persistence of fever, while only persistence of fever was the major reason of treatment failure in other trials [10,13]. The current study included a broader range of patients than the clinical trials, which could explain some of these differences. Among 7 patients who had positive fungal cultures prior to and after the empiric therapy, the treatment failure for these patients might be due to a low dose of fluconazole (6 patients on daily dose of 200 mg and 1 patient on 600 mg) while other trials on empiric fluconazole therapy applied a daily dose of 400 mg [10-13,19]. Hepatotoxicity was the major adverse event resulting in termination of fluconaozle [19], though it was rare [11,13]. The variation in mortality rates between studies may be due to differences in the severity of the underlying malignancies.

Several published studies have identified independent factors for clinical outcomes in cancer patients who had documented fungal infection [2,22,23]. or who received empiric antifungal therapy [11]. In these reports, independent correlates of death or persistent and breakthrough fungal infection in cancer patients included presence or persistence of neutropenia, increased severity of illness, visceral dissemination, previous use of corticosteroids, absence of antifungal treatment, older age and low performance status [2,22,23]. A trial comparing fluconazole and amphotericin B as empiric antifungal agents in cancer patients with prolonged fever and neutropenia examined the correlates of treatment failure and found that persistence of neutropenia and pneumonia were independent predictors [11]. However, in the current study, which included only oncology and bone marrow transplantation patients, patient age, duration of neutropenia and immunosuppressive therapy before initiation of fluconazole were not correlated with treatment failure. There was a univariate relationship between lower Charlson comorbidity score and treatment failure but this variable was not retained in the stepwise multivariate model.

In the current study, we found that treatment failure had a significant economic impact. Other recent studies have confirmed findings from earlier reports which demonstrated excess resource utilization attributable to fungal infections [24-30]. It might have been expected that patients with hematological malignancy would have longer duration of neutropenia and hospital stays resulting in higher treatment failure rates compared to patients with solid tumors. However, the economic costs associated with fungal infections and antifungal treatment in cancer patients have seldom been assessed, and in particular limited data are available from routine care. One study used data from cancer registries and Medicare claims to evaluate the resource utilization associated with fungal infections among hospitalized cancer patients and found that cancer patients with fungal infections had longer length of stay (10–11 days) and higher costs ($10,479–$15,966 in Medicare expenditures) compared with same age group of cancer patients without fungal infections [27]. The study by Menzin et al. also found that isolates of *Candida *species were the most common type of fungal infections. Similar findings on the baseline isolates were also found in the current study. However, neither details about antifungal medications nor the clinical response to antifungal treatment was reported by Menzin [27]. Another multicenter trial compared the hospital costs and length of stay between febrile neutropenic cancer patients on empiric therapy with liposomal and conventional amphotericin B, and found the mean total hospital costs to be $77,496 versus $82,075, and length of stay of 32.1 versus 32.5 days, respectively, although no data on treatment failure were reported [31].

Many clinicians believe that any cancer patient with persistent fever and profound neutropenia after 5 days of adequate doses of broad-spectrum antibiotics is a candidate for antifungal therapy [7]. Amphotericin B has been used as empiric antifungal agent for many years since the early efficacy data were published in 1980s [4,5]. However, serious toxicities associated with amphotericin B led to more randomized trials on empiric antifungal therapy using other agents including three lipid formulations of amphotericin B [20,32-34], azoles (fluconazole [10-13,35], itraconazole [36] and voriconazole [8]) and echinocandins (caspofungin [9]). The choice of agents for empiric antifungal therapy is not clear due to the similarity of clinical effectiveness found from above comparative trials, while decisions on specific antifungal drug use depends on multiple factors, including risk of infection caused by specific fungal pathogen, spectrum of activity, toxicity of drug, patient's tolerability to drug and treatment related costs [7,37-39].

The current study has several limitations. We used a retrospective design and relied on documentation in patient charts to identify clinically significant adverse events attributable to fluconazole. As a result, we may have missed undocumented adverse effects. The number of patients included in the final analysis may have been too small to identify some correlates of treatment failure. Finally, this study was performed in the inpatient setting at one tertiary care hospital, so the results may not be generalizable to other institutions or settings.

## Conclusion

The incidence of treatment failure associated with empiric fluconazole antifungal therapy is high among cancer patients, particularly in those suffering from hematological malignancies, and that the additional length of stay and hospital costs associated with treatment failure are high. These data suggest that alternative agents that reduce frequency of treatment failure may be cost-effective. Furthermore, additional evaluation to assess the frequency of treatment failure and its associated resource utilization should be carried out for other agents that are used for empiric antifungal therapy.

## Competing interests

DWB received research funding from Merck & Co., Inc; DTY received reimbursement from Merck & Co., Inc for attending a conference; RNK is employed by Merck & Co., Inc.

## Authors' contributions

DTY participated in study design, performed the statistical analyses and drafted the manuscript. DLS help to study design, performed the chart review and collected the data. JFP helped to study design and manuscript writing. RNK contributed to manuscript editing. DWB participated in all aspects of the study including study design, provision of study patients, data collection, data analysis and interpretation, manuscript writing. All authors read and approved the final manuscript.

## Pre-publication history

The pre-publication history for this paper can be accessed here:


